# Recent advances in microbial capture of hydrogen sulfide from sour gas via sulfur‐oxidizing bacteria

**DOI:** 10.1002/elsc.202100006

**Published:** 2021-06-03

**Authors:** Zheng Chen, Gama Yang, Xuemi Hao, Nadia A. Samak, Yunpu Jia, Sumit Peh, Tingzhen Mu, Maohua Yang, Jianmin Xing

**Affiliations:** ^1^ CAS Key Laboratory of Green Process and Engineering State Key Laboratory of Biochemical Engineering Institute of Process Engineering, Chinese Academy of Sciences Beijing P. R. China; ^2^ College of Chemical Engineering University of Chinese Academy of Sciences Beijing P. R. China; ^3^ Processes Design and Development Department Egyptian Petroleum Research Institute Cairo Egypt

**Keywords:** absorption enhancement, bioreactor modification, hydrogen sulfide, metabolic engineering, microbial capture, sulfur‐oxidizing bacteria

## Abstract

Biological desulfurization offers several remarkably environmental advantages of operation at ambient temperature and atmospheric pressure, no demand of toxic chemicals as well as the formation of biologically re‐usable sulfur (S^0^), which has attracted increasing attention compared to conventionally physicochemical approaches in removing hydrogen sulfide from sour gas. However, the low biomass of SOB, the acidification of process solution, the recovery of SOB, and the selectivity of bio‐S^0^ limit its industrial application. Therefore, more efforts should be made in the improvement of the BDS process for its industrial application via different research perspectives. This review summarized the recent research advances in the microbial capture of hydrogen sulfide from sour gas based on strain modification, absorption enhancement, and bioreactor modification. Several efficient solutions to limitations for the BDS process were proposed, which paved the way for the future development of BDS industrialization.

**Abbreviations**
ALRs: airlift reactors
BCRs: bubble‐column reactors
BDS: biological desulfurization
SGV: superficial gas velocity
SOB: sulfur‐oxidizing bacteria

## 1 INTRODUCTION

Hydrogen sulfide (H_2_S) must be removed from a considered amount of gas streams, including natural gas, biogas, refinery gas, synthesis gas, landfill gas, and tail gas, owing to its toxicity, corrosive properties, and unpleasant odor. Therefore, H_2_S containing gas demands treatment before its utilization to avoid industrial and environmental problems. Moreover, H_2_S has a harmful and even lethal effect on human health [1]. Generally, H_2_S is removed from sour gas by conventionally physicochemical desulfurization processes, such as liquid iron‐based technology, Amine‐Claus, Lo‐Cat, and SulFerox [2]. These technologies usually consume special chemicals and catalysts, perform at high temperature and pressure, and also require high investment or maintenance costs for industrial applications [3]. As a cost‐effective alternative to physicochemical desulfurization, H_2_S can be removed by biological desulfurization, which offers several remarkably environmental advantages of (i) operating at ambient temperature and atmospheric pressure, (ii) not requiring toxic and complex chemicals, and (iii) producing the biologically re‐usable sulfur (S^0^) [4, 5]. Moreover, BDS under haloalkaliphilic conditions is already proven to be effective in the removal and conversion of H_2_S from sour gas [5, 6]. At present, the BDS process has been widely applied on an industrial scale with over 250 installations worldwide in 2017 [6, 7], such as Shell‐Paques/THIOPAQ, which is a typical BDS technology based on mixed SOB (S^0^‐oxidizing bacteria) system, removing H_2_S from natural gas, biogas, synthesis gas, and refinery gas.[8]

**FIGURE 1:**
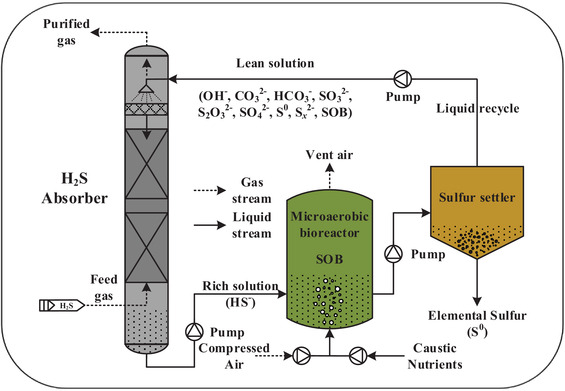
Schematic diagram of the biological gas desulfurization process. The biological desulfurization (BDS) process is composed of three parts, (I) the absorption of H_2_S gas, (II) the biological conversion of dissolved sulfide, (III) the separation and collection of bio‐sulfur

In the biological gas desulfurization process (Figure 1), the first step is to selectively absorb H_2_S from feed gas in an absorber column via countercurrent contact with an alkaline solution. The H_2_S gas dissolves into the liquid phase firstly (Equation 1), then hydroxide ions (OH^−^) and carbonate ions (CO2‐3) are consumed to react with dissolved H_2_S with the formation of bisulfide ion (HS^−^) (Equations 2 and 3) [9, 10, 11, 12, 13, 14, 15].

(1) $$\text{Physical}\;\text{absorption}\;H_{2}S\left(g\right)⇌H_{2}S\left(\text{aq}\right)$$

(2) $$\text{Chemical}\;\text{absorption}\;H_{2}S\left(\text{aq}\right)+OH^{-}⇌HS^{-}+H_{2}O$$

(3) $$\;\text{Chemical}\;\text{absorption}\;H_{2}S\left(\text{aq}\right)+{\text{CO}}_{3}^{2-}⇌HS^{-}+{\text{HCO}}_{3}^{-}$$

The absorber solution is subsequently routed to an aerated microaerophilic bioreactor, where HS^−^ is mainly oxidized to elemental sulfur (S^0^) by SOB under oxygen‐limited conditions (Equation 4) [16, 17, 18, 19]. And the bioreactor solution is recycled to the absorber column, containing (bi) carbonate, bio‐S^0^ particles, SOB, and (thio)sulfate. Meanwhile, a part of the bioreactor solution, containing the bio‐S^0^ particles, is lastly fed to the S^0^ separation section, the bio‐S^0^ is obtained by gravity settling or centrifugation.

(4) $$\mathrm{Biological}\;\mathrm{sulfide}\;\mathrm{oxidation}\;2HS^{-}+O_{2}\to 2S^{0}+2OH^{-}$$

Also, unfavorable side reactions involving HS^−^ ion also take place, including HS^−^ is completely biologically oxidized to SO2‐4 under HS^−^ limited conditions (Equation 5) [16, 17, 18, 19], HS^−^ is oxidized chemically to the thiosulfate (S_2_O_3_^2‐^) (Equation 6) [16, 17, 18], and the formation of polysulfide ions (S_x_^2‐^) is owing to the reaction between HS^−^ and S^0^ (Equation 7) [9, 10, 12, 14]. Polysulfide ions (S_x_^2‐^) are chemically oxidized to S^0^ and S_2_O_3_^2‐^ in presence of dissolved O_2_ (Equation 8) [9, 12, 20].

(5) $$\mathrm{Biological}\;\mathrm{sulfide}\;\mathrm{oxidation}\;HS^{-}+2O_{2}\to {\mathrm{SO}}_{4}^{2-}+H^{+}$$

(6) $$\begin{array}{ccc} \mathrm{Chemical}\;\mathrm{sulfide}\;\mathrm{oxidation}\;HS^{-} & + & O_{2}\to 1/2S_{2}O_{3}^{2-}\\ & + & 1/2H_{2}O \end{array}$$

(7) $$\begin{array}{ccc} \text{Chemical}\;\text{polysulfide}\;\text{formation}\;HS^{-} & + & \left(x-1\right)S^{0}⇌\\ & & S_{x}^{2-}+H^{+} \end{array}$$

(8) $$\begin{array}{ccc} \mathrm{Chemical}\;\mathrm{polysulfide}\;\mathrm{oxidation}\;S_{x}^{2-} & + & 1/2O_{2}\to S_{2}O_{3}^{2-}\\ & + & \left(x-2\right)S^{0} \end{array}$$

Therefore, as we know, the formation of SO_4_^2‐^ and S_2_O_3_^2‐^ results in less production of bio‐S^0^ and higher O_2_ demand. Moreover, it also leads to the acidification of the process solution, increasing the demand for an alkaline solution [9].

**PRACTICAL APPLICATION**

The BDS process has successful and promising applications in removing H_2_S from sour gas, such as natural gas and biogas, because of the significant environmental advantages compared to conventional physicochemical processes. However, the low biomass of SOB, the acidification of the process solution, the recovery of SOB, the absorption efficiency of H_2_S, and the performance of the bioreactor are challenges and restrictions for practical manufacturing and industrial applications of the BDS process. Therefore, it is necessary to settle these difficulties to achieve maximum exploitation of the BDS process for industrial applications. Herein, this review highlights recent research progress on improving the BDS process, especially discussing various strategies to solve these challenges from perspectives of strain modification, absorption enhancement, and bioreactor modification, which help us obtain deep insight into improving industrial applications of the BDS process and provide future research directions for developing efficient biological desulfurization.

The biologically produced S^0^ (bio‐S^0^), the end product of the bio‐desulfurization process, differs distinctly from crystalline elemental S^0^, particularly in possessing more hydrophilic properties and dispersing in an aqueous solution since that the surface of S^0^ globules is coated with a vesicle structure composed of bio‐macromolecules, such as proteins and polysaccharides (SgpA, SgpB, and SgpC proteins in *Allochromatium vinosum*) [21]. Bio‐S^0^ is formed in particles with dimensions of around 0.1–1 μm, these particles can form aggregates of elemental S^0^ with dimensions up to 3 mm. Crystalline chemical S^0^ is strongly hydrophobic and poorly soluble in an aqueous solution compared to bio‐S^0^. Moreover, the major structure of bio‐S^0^ is rings of 8 S‐atoms (S_8_), which is composed of the core of S^0^ with organic polymers adsorbed on the particle surface, providing steric stabilization and electrostatic repulsion against aggregation. As a result of the small size (micro‐sized and nano‐sized) and hydrophilic surface, bio‐S^0^ has better applications than chemical S^0^ in bioleaching and fertilizer [22, 23, 24, 25]. Because of the size and surface of nano‐sized S^0^, it can be used for various biomedical industries. Furthermore, nano‐sized S^0^ is also used to modify carbon nanotubes and synthesize nanocomposites for lithium‐S^0^ batteries [26, 27, 28]. More importantly, nanosized sulfur's price is 25 ∼ 40 fold higher than microsized S^0^ due to their complicated production procedures and superior characteristics. Nanometric (>50 nm) S^0^ bioproduction has been confirmed by the bioconversion of H_2_S from sour gas using *Thioalkalivibrio versutus* at haloalkaliphilic conditions [25]. Therefore, the BDS process is a cost‐effective and mutually beneficial strategy for producing nano‐sized S^0^ and removing H_2_S from sour gas. Hence, modifying and optimizing the bio‐desulfurization process to increase the selectivity of S^0^ formation, improve the production of S^0^, and form more effective nanosized S^0^ becomes necessary and meaningful.

Nevertheless, several limits and difficulties involving SOB strains, absorber, bioreactor, and process control still exist in the BDS process, such as (i) SOB is a non‐model organism with low biomass and no efficient genetic manipulation tools [29], which hinder the improvement of their beneficial uses via genetic engineering [30]; (ii) the production of thiosulfate (S_2_O_3_^2^^‐^) and sulfate (SO_4_^2^^‐^) during the oxidation process easily cause the acidification of the process solution, so alkaline solution needs to be supplied into liquid phase, which increases the costs [31]; (iii) the loss of SOB biomass happens during the bio‐S^0^ separation process since SOB are integrated with bio‐S^0^ [32]; (iv) the absorption of H_2_S in the absorber demands to be improved because it directly determines the residual H_2_S in purified gas, moreover dissolved H_2_S entering into the bioreactor is poisonous for SOB [33]; (v) there is a lack of the preliminary mathematical modeling of the complicated phenomena occurring in the bioreactors to study the main reaction parameters of bioreactors, even though several experimental studies have been performed to explore the performance of the bioprocess [34].

Fortunately, there have been several studies focused on making the BDS process better concerning these puzzles. Here, we reviewed some relevant research work and summarized three parts about how to make it better, listed as following:

- Microorganism section: Increase the biomass of S^0^‐oxidizing bacteria via feeding strategies; avoid the acidification of process solution via several methods; accumulate the production of bio‐S^0^ and avoid by‐products through genetic and metabolic modification; solve recycling problems of SOB via immobilization methods.
- Absorber section: Enhance the absorption of H_2_S in the absorber column via chemical and biological approaches.
- Bioreactor section: Improve the mass transfer in bioreactor via design various geometries and figure out the flow dynamics characteristics in bioreactor via numerical simulation for reactor design, optimization, and scale‐up.

## 2 SOB: A POTENTIAL CANDIDATE FOR THE CAPTURE OF H_2_S

S^0^‐oxidizing bacteria play a major role in the microbial S^0^ cycle, reduced S^0^ compounds can be oxidized to elemental S^0^ or sulfate. Therefore, the microbial S^0^ cycle offers substantial opportunities for the treatment of polluted gas and wastewater in the environmental technology and process industry [22]. SOB grow with inorganic carbon (CO_2_) as a carbon source and obtain chemical energy from the oxidation of reduced inorganic compounds. Generally, biodegradation of H_2_S via SOB occurs under (micro) aerobic conditions with O_2_ as an electron acceptor, or under anaerobic conditions with alternative electron acceptors, which depends on different SOB [35].

Thus far, acidophilic, neutrophilic, and alkaliphilic SOB have been intensively investigated, and their characteristics are presented in Table 1 [36, 37, 38, 39, 40, 41, 42, 43, 44]. These desulfurization microorganisms are obligate chemoautotrophs that can convert reduced S^0^ species, such as elemental S^0^, sulfide, and thiosulfate, to intracellular/extracellular S^0^ globules via oxidation. They can be widely applied in various fields owing to that they can grow at a wide range of pH and temperature. Many *Thiobacillus* sp., such as *Thiobacillus thiooxidans* and *Thiobacillus ferrooxidans*, have acidophilic characteristics and have the ability to grow under acidic conditions of low pH. *Thiobacillus thiooxidans* has a great tolerance for acidic conditions and can grow at pH <1. *Thiobacillus ferrooxidans* plays the dominant role in the bio‐extractive process because they can oxidize both iron and reduced S^0^ compounds under acidic conditions. Other *Thiobacillus sp*. (e.g., *Thiobacillus denitrificans, Thiobacillus thioparus*, and *Thiobacillus neapolitanus*) can develop in the neutral medium at pH of 6–8, *T. denitrificans* and *T. neapolitanus* have been used in Shell‐paques bio‐desulfurization technology for removing H_2_S under weakly alkaline conditions. Other strains can degrade S^0^ compounds under haloalkaliphilic conditions, such as *Thioalkalispira microaerophila*, *Thioalkalivibrio versutus*, and *Thioalkalimicrobium aerophilum*, isolated from soda lakes, can grow at high pH and salinity, which makes it suitable for the bio‐desulfurization process. *Thioalkalispira microaerophila* can develop at alkaline conditions and attains optimum growth at pH 10, but it forms intracellular S^0^, which hinders its industrial applications because it is not easy to separate. In addition, these chemoautotrophs can degrade S^0^ compounds under various environmental conditions and are not limited by light source compared to photoautotroph, as a result, SOB is the most widely used desulfurization bacteria.

**TABLE 1:** Characteristics of sulfur‐oxidizing bacteria implicated in the degradation of H_2_S

	Acidophilic			Neutrophilic			Alkaliphilic		
Characteristics	*Thiobacillus ferrooxidans*	*Thiobacillus thiooxidans*	*Thiomicrospra frisia*	*Thiobacillus denitrificans*	*Thiobacillus thioparus*	*Thiobacillus neapolitanus*	*Thioalkalispira microaerophila*	*Thioalkalivibrio versutus*	*Thioalkalimicrobium aerophilum*
pH growth range	1.5‐6.0	0.5‐6.0	4.2‐8.5	–	5.0‐9.0	–	8.0‐10.4	7.5‐10.65	7.5‐10.6
Optimum pH	2.0	2.0‐3.5	6.5	6.8‐7.4	7.5	7.0	10.0	10.0‐10.2	9.5‐10.0
Temperature growth range (°C)	2‐37	10‐37	3.5‐39	–		–	–	<50	<39
Optimum temperature (°C)	30	28‐30	32‐35	28‐32	28	30	–	–	–
G+C content of DNA (mol%)	56.3‐58.8	52.0‐62.0	39.6	63.0	62.0	–	58.9	61.0‐66.5	47.3‐51.2

Oxygen requirement	Facultative anaerobe	Strictly aerobe	Strictly aerobe	Facultative anaerobe	Strictly aerobe	Strictly aerobe	Strictly (micro)aerobe	Strictly aerobe	Strictly aerobe
Examples of energy source	Ferrous ion, reduced sulfur compounds	Hydrogen sulfide, elemental sulfur	Thiosulfate, tetrathionate, elemental sulfur, sulfide	Thiosulfate, tetrathionate, thiocyanate, elemental sulfur, sulfide	Thiosulfate, tetrathionate, thiocyanate, trithionate, sulfide	Thiosulfate, tetrathionate, sulfite, sulfide, elemental sulfur	Thiosulfate, polysulfide, elemental sulfur, sulfide,	Thiosulfate, Trithionate, tetrathionate, polysulfide, elemental sulfur, sulfide,	Thiosulfate, tetrathionate, sulfide, polysulfide, elemental sulfur

Sulfur deposit	–	–	Extracellular	–	Extracellular	–	Intracellular	Extracellular	Extracellular
Cell type	Gram‐negative								

Trophy	Obligate chemoautotroph								
References	[36]	[37, 38]	[39]	[40]	[41]	[42]	[43]	[44]	[44]

## 3 STRATEGIES FOR IMPROVING THE MICROBIAL CAPTURE OF H_2_S USING SOB

**FIGURE 2:**
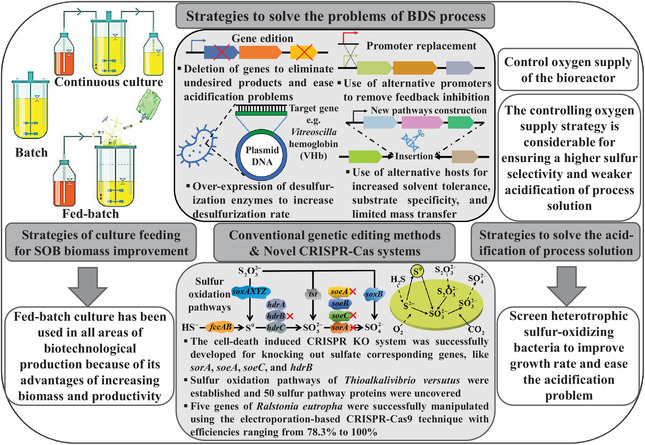
Schematic diagram summarizing reported strategies to solve mentioned above problems in the biological desulfurization (BDS) process. There are four parts of solving problems that appeared in the BDS process, including (I) the biomass of SOB is improved via fed‐batch strategy, (II) controlling O_2_ supplied to the bioreactor and screening heterotrophic sulfur‐oxidizing bacteria (SOB) are beneficial to ease the acidification of process solution, (III) metabolic engineering of SOB is achieved by conventional genetic editing methods, including genes deletions, over‐expression enzymes, use of alternative promoters, and use of alternative hosts, (IV) novel CRISPR‐Cas systems are used in gene‐editing of SOB

As we know, SOB is a fundamental and significant component of the biological gas desulfurization process, directly influencing the oxidization rate of sulfide and the yield of bio‐S^0^. Therefore, improving the biomass and activity of SOB, reducing undesired products (sulfate and thiosulfate), and accumulating bio‐S^0^ are meaningful and arduous work. Representative difficulties in the BDS process include the acidification of process solution due to excessive oxidation of sulfide, low SOB biomass and desulfurization activity, recycling problems of SOB. A summary of reported strategies to solve the mentioned above problems has been presented in Figure 2.

### 3.1 Fed‐batch strategy for SOB biomass improvement

Substrates supply is necessary for any bioprocess, whether added at the beginning or provided over time based on several strategies. Because substrates supply affects the biomass, as a result, product yield and bioprocess cost are influenced. Batch, fed batch, and continuous culture are used to cultivate microorganisms in the bioreactor, and which culturing method to use depends on the microorganism, application, and final goal, as following: (i) Batch is appropriate for the bioprocess of fast cultivation and limited time‐consumed, (ii) fed‐batch is suitable for the bioprocess of high cell density and flexible productivity, (iii) continuous culture is proper for the stable and enduring bioprocess [45].

Normally, fed‐batch culture is easier to control concentrations of substrates compared to batch culture, because it affects the desired metabolite's yield or productivity. Moreover, fed‐batch culture has been used in various fields of biotechnological production due to its advantages of increasing growth and biomass [46, 47, 48, 49]. Sharshar et al. found that fed‐batch culture resulted in higher biomass of *Thioalkalivibrio versutus* and productivity of bio‐S^0^ compared to batch culture [29]. Also, Guajardo et al. discovered that the fed‐batch bioreactor had a higher conversion compared to the batch bioreactor [49].

To obtain better performance of fed‐batch culture, more attention should be paid to the feeding strategy, which is generally based on time, pH, and dissolved oxygen [47]. For instance, Sharshar's fed‐batch strategy was based on time, which enhanced biomass and bio‐S^0^ productivity. Nevertheless, the time‐based feeding strategy is complicated, because the feeding substrate was not accurate at a different time. Therefore, we should investigate an easier and more efficient feeding strategy based on fed‐batch culture to improve SOB biomass by measuring the maximal growth rate, O.D_600_ value, biomass, and bio‐S^0^ yield. In the BDS process, the desulfurization capability of SOB is affected by reaction conditions of bioreactors, such as pH, ORP (oxidation‐reduction potential), Na^+^ concentration, and growth temperature, so we tend to employ different feeding strategies based on different reaction parameters by feeding air, caustic solution, and nutrients in batch to control suitable reaction conditions.

### 3.2 Strategies to solve the acidification of process solution

Generally, adequate oxygen supplied to the bioreactor confirms the complete conversion of dissolved sulfide, which results in incomplete oxidation of sulfide to elemental S^0^ and complete oxidation of sulfide to unfavorable (thio) sulfate. Nonetheless, thiosulfate (S_2_O_3_^2‐^) and sulfate (SO_4_^2‐^) cause the acidification of the process solution. However, most SOB can tolerate alkaline conditions, so caustic are required to maintain the alkalinity of the process solution, which leads to a higher running cost [50, 51, 52]. Moreover, the acidified process solution is also not beneficial to the absorption of H_2_S in the absorber column.

Fortunately, controlling O_2_ supplied to the bioreactor is an efficient solution to weak acidification [53, 54, 55]. According to Equation 4, the yield of bio‐S^0^ is maximal when O_2_/H_2_S supply molar ratio is 0.5. In fact, the maximal S^0^ productivity was obtained at a slightly higher O_2_/H_2_S supply molar ratio [12, 56]. Thus, the controlling O_2_ supply strategy is considerable for ensuring a higher S^0^ productivity and weaker acidification of process solution.

Recently, a feed forward control strategy based on O_2_/H_2_S supply ratio was proposed for controlling O_2_ supplied to the bioreactor, which was considered as an alternative to the feedback control strategy based on ORP because of its reliable, stable, and robust performance when it was applied in the BDS process. Nevertheless, this strategy has a limiting factor, the sulfide feed concentration to the BDS system is required to be determined. Thus, the H_2_S detector needs to be installed on the feed gas line to control O_2_ supplied to the bioreactor based H_2_S feed concentration [57].

Besides, screening heterotrophic S^0^‐oxidizing bacteria is another solution to the acidification problem. Hou et al. found that several heterotrophic bacteria with sulfide:quinone oxidoreductase (SQR) and persulfide dioxygenase (PDO) cultured with organic compounds could convert H_2_S to S^0^ and S_2_O_3_^2‐^ during the oxidization process, the pH in the liquid phase was slightly changed after sulfide oxidation at the same time [31]. Therefore, heterotrophic SOB may be used as an alternative to chemolithoautotrophic SOB for microbial capture of H_2_S because of its advantages of fast growth and no apparent acidification. However, an additional organic carbon source needs to be supplied to the bioreactor when heterotrophic SOB are employed for H_2_S removal in the BDS process.

### 3.3 Metabolic engineering and genome editing of SOB

Desulfurization bacteria is a crucial part of the BDS process, such as *Thioalkalivibrio versutus*, *Thiobacillus ferrooxidans*, and *Thiobacillus denitrificans*, which are chemolithoautotrophic SOB. SOB with high desulfurization activity and stability are the prerequisite for the industrialization of biological gas desulfurization [58].

Consequently, related researchers focused on enhancing the desulfurization activity of SOB and solving the acidification of process solution utilizing genetic manipulations of the desulfurization pathway based on conventional genetic engineering methods [59], including (i) over‐expression of desulfurization enzymes to increase desulfurization rate, (ii) use of alternative promoters to remove feedback inhibition, (iii) deletion of genes to eliminate undesired products and ease acidification, (iv) use of alternative hosts for increased solvent tolerance, substrate specificity, limited mass transfer, and thermostability, like *Escherichia coli*, which is the significant industrial strain used in producing various biochemicals and biofuels, including 1,4‐butanediol, fatty acids, and aromatic polyesters [60, 61, 62, 63]. Xin et al. used *E. coli* as recombinant to analyze functions of SQR and PDO genes, it was found that recombinant *E. coli* with SQR and PDO rapidly oxidized sulfide to S_2_O_3_^2‐^ and SO_3_^2‐^ [64]. *Pseudomonas aeruginosa* and *Saccharomyces cerevisiae* were also reported, the recombinant (*P. aeruginosa*) with flavocytochrome c sulfide dehydrogenases (FCSDs) rapidly oxidized sulfide to S^0^ and the recombinant with FCSD and PDO oxidized sulfide to S_2_O_3_^2‐^ and SO_3_^2‐^ [65], the pathway for thiosulfate utilization in *S. Cerevisiae* was deciphered.[66]

And a higher desulfurization rate was obtained via the above methods. However, the enhanced desulfurization rate did not fulfill industrial requirements of the BDS process and conventional methods of genome editing have certain limitations. Therefore, it requires stable and precise gene‐editing tools for genetic manipulations of SOB to improve the bio‐desulfurization activity and efficiency of the BDS process.

Clustered regularly interspaced short palindromic repeats (CRISPR) and CRISPR‐associated (Cas) proteins constitute an adaptive immune system against phages and other foreign genetic elements in bacteria and archaea [67]. CRISPR‐Cas systems possess several superiorities of high efficiency, handling easily, specificity and robustness compared to previously used genetic engineering tools, which have been successfully used in various microorganisms, including many industrially important bacteria [59]. Moreover, they have been used to manipulate the genome of desulfurization bacteria, such as *Thioalkalivibrio versutus* and *Ralstonia eutropha*, to increase the yield of desired products.

*Thioalkalivibrio versutus* is a kind of autotrophic and gram‐negative bacteria, which can convert sulfide to bio‐S^0^ or sulfate at haloalkaliphilic conditions. Like other autotrophs, it also lacks precise and efficient genome editing tools and uncovered metabolic pathways, which hamper increasing activity and efficiency of bio‐desulfurization [30]. Recently, Mu et al. established a highly efficient genetic transformation system based on the conjugative transfer method in the genus *Thialkalivibrio*. Meanwhile, the average thiosulfate removal rate of *T. versutus* SOB306 was improved 11.7 ± 1.8% by expressing *Vitreoscilla* hemoglobin (VHb), because it utilizes dissolved O_2_ more efficiently with the help of VHb [68]. With further research, Sharshar et al. established precise *T. versutus* S^0^ oxidation pathways by comparative genomics and transcriptome analysis, and 50 S^0^ pathway proteins were uncovered using protein mining and transcriptome studies for finding sulfate producing genes [25]. Amongst identified S^0^ proteins, genes of sulfate metabolic pathway (*sorA*, *soeA*, *soeC*, and *hdrB*) were an explanation for sulfate formation. In order to lower sulfate production, a tight Ferrous ion‐inducible expression system for *T. versutus* was successfully developed to control sulfate corresponding gene expressions such as *hdrABC*, *sorA*, and *soeABC* [29]. Meanwhile, the cell‐death induced CRISPR KO (knockout) system was successfully developed for knocking out sulfate corresponding genes. Further, whether knocking out the *hdrB* gene influences sulfate production was studied, and experimental results demonstrated using the Δ*hdrB* mutant strain, its decreasing percentage of sulfate production was 55.1%, and elevating percentage of S^0^ production was 166.7% compared to the native strain while using sulfide as an energy source. Also, the Δ*hdrB* mutant strain grew faster than the native strain verified by its doubling time, maximum growth rate, and maximum OD_600_ values [25]. As a consequence, knocking out the *hdrB* gene of *T. versutus* employing cell‐death induced CRISPR KO system is an effective and reliable strategy of improving S^0^ production and lowering sulfate production, but it still demands industrial application to confirm the feasibility of the application of mutant stains.

Five genes of *Ralstonia eutropha* were successfully manipulated using an electroporation‐based CRISPR‐Cas9 technique with efficiencies ranging from 78.3% to 100%, which significantly increased the efficiency and decreased time to manipulate this facultative chemolithoautotrophic microbe [69]. And *Ralstonia eutropha* has been reported as a competent desulfurization biocatalyst for DBT (dibenzothiophene) desulfurization [70, 71]. Therefore, there have been several successful studies in establishing genome editing techniques for genome editing of desulfurization bacteria based on CRISPR‐Cas systems, which paves a way for future CRISPR Cas systems to enhance bio‐S^0^ production and lessen sulfate production in the biological gas desulfurization process.

### 3.4 Immobilization of SOB: toward a cost‐effective desulfurization process

The SOB microorganisms are inconvenient to be recovered because of their small cell size and electrical stability [72, 73]. In the bioreactor of the BDS process, a considerable amount of bio‐S^0^ is integrated with SOB and then fed to the separation section, which easily results in the loss of SOB [32]. Therefore, the recycling of SOB is a limiting factor of the bio‐desulfurization process. Fortunately, SOB immobilization can help the BDS process obtain higher SOB concentration and desulfurization efficiency. Compared to entrapment and adsorption, magnetic immobilization has more wide applications, mainly because it has a small mass transfer limitation of substrate diffusion into the reaction system [74, 75].

Recently, magnetic Fe_3_O_4_ nanoparticles have been applied to immobilize and separate haloalkaliphilic SOB (*Thialkalivibrio versutus* D301), the results demonstrated that the immobilization method had no effect on sulfide oxidation capacity, and immobilized cells were reused at least six times. [32]. Furthermore, Mu et al. utilized Fe_3_O_4_ nanoparticles modified with 3‐aminopropyl‐triethoxysilane to immobilize *T. versutus* D301, and reuse times of SOB were further increased [73]. Thus, using magnetic nanoparticles to immobilize SOB is beneficial to bio‐desulfurization and further research seems necessary to optimize its industrial application, like developing more stable and efficient immobilizing materials or verifying the effect of immobilized SOB in the large‐scale bioreactor.

## 4 ENHANCED ABSORPTION OF H_2_S IN THE ABSORBER COLUMN

**FIGURE 3:**
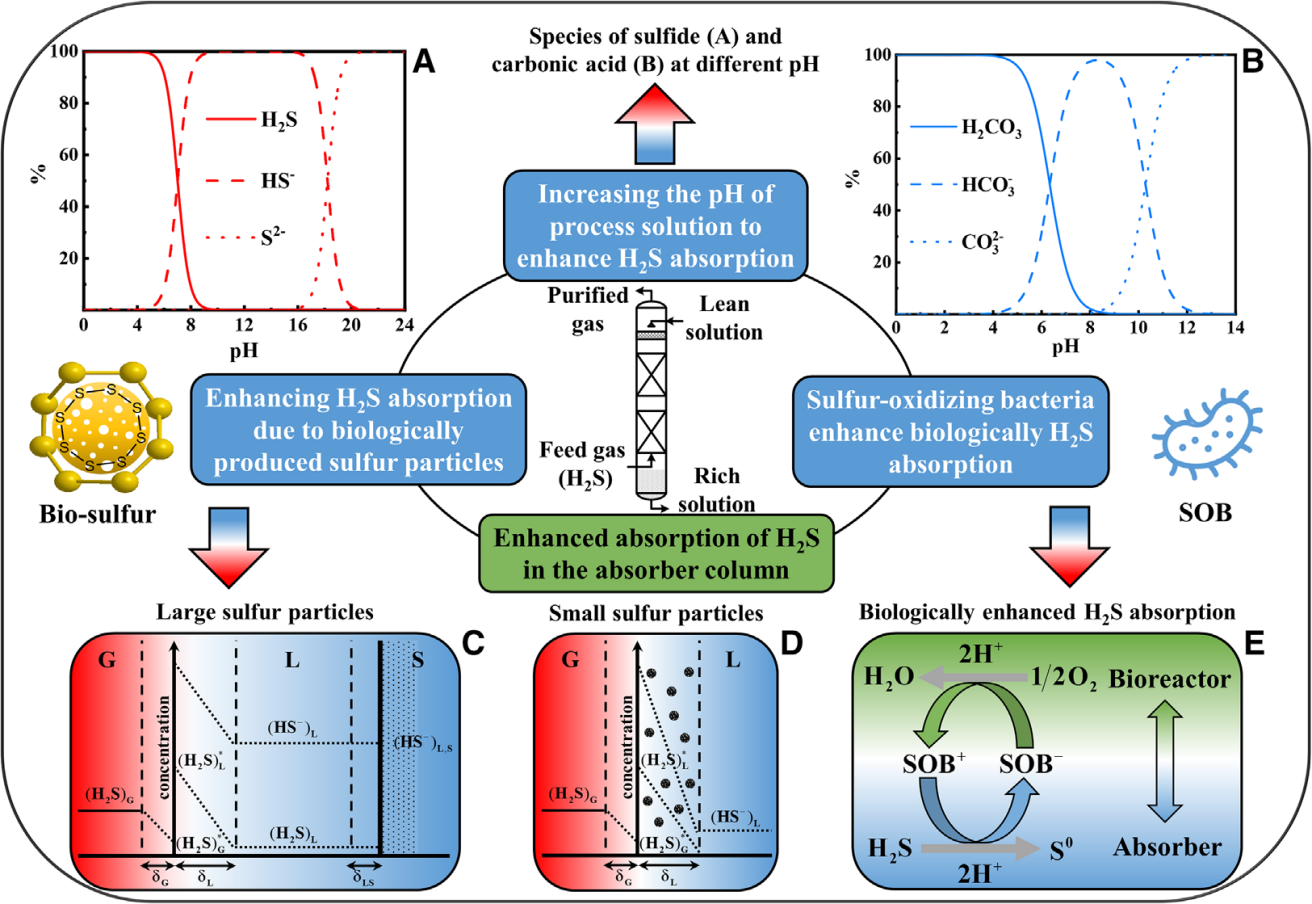
Schematic diagram summarizing methods of H_2_S absorption enhancement in the absorber column. (A, B) The species of hydrogen sulfide (H_2_S) and carbonic acid at different pH. (C, D) The concentration profiles of H_2_S and HS^−^ in gas‐liquid mass transfer for large and small sulfur particles. (E) The mechanism of biological enhanced H_2_S absorption

As mentioned above, after feed gas entered into the absorber column, H_2_S gas dissolved in the liquid phase and then reacted with alkaline carbonate solution (OH^−^ and CO_3_^2‐^) to form dissolved sulfide (HS^−^). The absorption step affects directly the removal rate of H_2_S and the residual H_2_S concentration of the purified gas [6]. Moreover, excessive dissolved H_2_S in the bioreactor has a poisonous effect on SOB, even causing the inactivation of SOB, which is damaging for the BDS process [33]. Thus, increasing the mass transfer rate in the H_2_S absorption process is a necessary and meaningful work for the biological gas desulfurization process. Several chemical and biological approaches to strengthening H_2_S absorption for biological gas desulfurization were presented in Figure 3.

### 4.1 Increasing the pH of process solution to enhance H_2_S absorption

In the absorber column, we all know, the occurred chemical reactions are mainly the homogeneous reactions of dissolved H_2_S with OH^−^ and CO_3_^2‐^, forming dissolved sulfide (HS^−^). From the perspective of chemical absorption reactions, a higher pH is in favor of improving H_2_S mass transfer. The mentioned above is the first enhancement mechanism of H_2_S absorption. Generally, the enhancement of H_2_S absorption is quantified as the enhancement factor, which is dependent on the pH of the process solution [76].

The H_2_S dissolves into the solution as a weak acid and then occurs secondary dissociation, forming HS^−^ and S^2−^. The p*K*a values of the second dissociation (H_2_S) are varying in literature from 13 to 19 [77] and the most reliable p*K*a^2^ value is 17.4 [78]. Therefore, S^2−^ could be considered as an insignificant species in an aqueous solution. But the concentration of H_2_S species strongly depends on pH, as shown in Figure 3A [79]. In the same way, the species of carbonic acid at different pH is presented in Figure 3B.

Therefore, under the conditions in the biological gas desulfurization system (i.e., around pH 9.0, which is a typical characteristic of the BDS system), the main components in the process solution are dissolved bisulfide (HS^−^), bicarbonate (HCO‐3), and carbonate (CO_3_^2‐^). Amongst these components, there is an equilibrium reaction between bicarbonate (HCO_3_^‐^) and hydroxide (OH^−^) ions, which equals Equation 3–Equation 2. Therefore, the pH of the process solution is relatively constant because of the buffer solution system.

The chemical enhancement of H_2_S absorption depends on the pH and alkalinity of the process solution, but it does not mean that the higher pH is beneficial to H_2_S absorption, because SOB have the limitation of pH tolerance. Further, now many researchers are trying to screen special strains from natural habitats, which can tolerate higher pH and salinity. Fortunately, haloalkaliphilic S^0^‐oxidizing bacteria (HA‐SOB) isolated from soda lakes can grow optimally at around pH 10 in media strongly buffered with sodium carbonate/bicarbonate and cannot grow at pH <7.5 and Na^+^ concentration <0.2 M, like *Thioalkalimicrobium, Thioalkalivibrio* and *Thioalkalispira* [44]. Under conditions of around pH 10, the mainly existing form of sulfide in aqueous solution is HS^−^, not like H_2_S, it cannot freely cross the cell membrane and thus HS^−^ is not toxic to SOB [33]. Hence, based on HA‐SOB, we could increase the alkalinity of the process solution to chemically enhance H_2_S absorption.

### 4.2 Enhancing H_2_S absorption due to biologically produced S^0^ particles

The gas‐liquid mass transfer rate was enhanced because of the existence of solid particles, this phenomenon had been reported, and five cases of enhancing gas‐liquid mass transfer as following: (i) physical adsorption on small particles, (ii) fast homogeneous reactions due to inert particles, (iii) homogeneous reaction with dissolving particles, (iv) reactive particles, and (v) heterogeneous reaction with catalytic particles [80].

For case (iii), we knew that H_2_S absorption was enhanced via increasing the pH of the process solution. For case (v), Mehra and Sharma found that H_2_S absorption rate in potassium iodide solution was enhanced and they considered that it was caused by the interaction of dissolved H_2_S and S^0^ particles in an aqueous solution [81]. Demmink et al. found that the absorption rate of acetylene was enhanced considerably due to the existence of S^0^ particles [82].

Mechanisms of enhanced gas absorption caused by the presence of particles have been investigated. Some researchers considered that particles act as shuttles, which means that adsorption of dissolved gas on the particle surface occurs in the diffusion layer, and after diffusion of the particle to the bulk, desorption occurs, they called this mechanism “grazing or shuttle effect” [83, 84]. Other researchers considered the enhancement of gas absorption depends on two effects, (i) accumulation of particles at the gas/liquid interface and (ii) a high absorption capacity of the particle for the dissolved gas, they called it particle‐at‐interface models [82, 85]. Besides, Sada et al. believed that SO_2_ absorption in the solution of Ca(OH)_2_ particles was enhanced because of the heterogeneous reaction between SO_2_ and Ca(OH)_2_ [86]. Moreover, this mechanism of enhanced gas absorption is established only when the heterogeneous reaction is fast and the size of particles is smaller than the gas/liquid film thickness [11].

The particle‐at‐interface models assume that enhancement of gas absorption only occurs in hydrophobic particles, which accumulated at the gas/liquid interface, supported by several experiments about enhancing gas absorption [82, 85]. As mentioned above, biologically produced S^0^, not like hydrophobic crystalline elemental S^0^, are hydrophilic and soluble in an aqueous solution [23]. Bio‐S^0^ particles with dimensions of approximately 0.1∼1 μm can form aggregates of bio‐S^0^ with dimensions up to 3 mm [23]. Thus, small bio‐S^0^ particles are possibly too hydrophilic to be located at the gas/liquid interface and large bio‐S^0^ particles are probably somewhat more hydrophobic, causing larger fraction not to be located at the gas/liquid interface [24]. Also, the heterogeneous reaction of dissolved sulfide with elemental S^0^ (Equation 7) may be an explanation for the enhancement of H_2_S absorption due to the presence of bio‐S^0^ particles.

Whether bio‐S^0^ particles have an enhancing influence on H_2_S absorption in the BDS process and which mechanism could be an explanation for enhanced H_2_S absorption was investigated by Kleinjan et al. [11]. They found that the presence of bio‐S^0^ particles enhances the rate of H_2_S absorption in the BDS process. Also, they proposed that the mechanism of this enhancement depends on the type of particles. For hydrophilic and small (*d*_p_ <3 μm) biologically produced S^0^ particles, the heterogeneous reaction between S^0^ and HS^−^ can be an explanation for the enhancement of H_2_S absorption. For larger (*d*_p_ up to 20 μm) bio‐S^0^ particles; however, an increased rate of H_2_S absorption is probably explained by the more hydrophobic behavior of the particles resulting in a local increase of the hydrophobic S^0^ particle concentration near the gas/liquid interface and specific adsorption of H_2_S at the particle surface. In a three‐phase system, concentration profiles of H_2_S and HS^−^ in gas‐liquid mass transfer with a heterogeneous reaction are schematically shown in Figure 3C and 3D. For large S^0^ particles, H_2_S transfers from gas to liquid firstly and then from liquid to solid with the heterogeneous reaction of HS^−^ on the liquid/solid interface. For small S^0^ particles, mass transfer and heterogeneous reaction occur simultaneously in the liquid film [11].

### 4.3 S^0^‐oxidizing bacteria enhance biologically H_2_S absorption

Since that sulfide can be removed by SOB under anaerobic conditions [6, 87], whether the SOB have an enhancing effect on H_2_S absorption was investigated by de Rink et al. [15]. They discovered that increasing biomass concentration of SOB in the lean solution to the top of the absorber column, the residual H_2_S concentration in the treated gas decreased. Hence, they had a conclusion that SOB increase the absorption efficiency of H_2_S. In other words, SOB can biologically enhance H_2_S mass transfer in the absorber column, but not like physico‐chemical enhancement (i.e., homogeneous reactions of dissolved H_2_S with OH^−^ and CO_3_^2‐^, the heterogeneous reaction of HS^−^ with S^0^), biological enhancement is susceptible to physiological parameters, such as the community composition and gene expression levels of SOB [15]. Therefore, the biological enhancement of H_2_S absorption in the absorber column depends on the biomass concentration of SOB.

Further, the mechanism of biologically enhanced H_2_S absorption was investigated. Rink et al. considered that SOB probably oxidized sulfide under anaerobic conditions in the absorber, more details presented in Figure 3E. They assumed that SOB reach a lower oxidation state in the absorber due to the conversion reactions of sulfide. Subsequently, oxidation of SOB takes place via reduction of oxygen under aerobic conditions in the bioreactor. Therefore, electrons harvested by SOB from sulfide oxidation in the absorber can be transferred to the bioreactor and then loss electrons via reduction of oxygen because of the circulated process solution containing SOB between absorber and bioreactor, which results in the biological enhancement of H_2_S absorption [15]. Nonetheless, the mechanism of biological enhancement has not been fully understood till now, maybe future work should focus on potential kinetics and reaction pathways.

## 5 MODIFICATION, OPTIMIZATION, AND SIMULATION OF BIOREACTOR

One of the key engineering issues in the development of the gas bio‐desulfurization process is reactor design. To date, many bioreactors for bio‐desulfurization have been invented, and outstanding achievements have been obtained. The bio‐desulfurization process involves gas‐liquid‐solid phases. It is well recognized that airlift reactors (ALRs) are a typical multi‐phase pneumatic agitated reactor for bio‐desulfurization process due to simple construction without moving parts, excellent interphase contacts, low shear stress, well‐defined fluid flow, high mass/heat transfer performance, and low operational energy consumption [88, 89, 90, 91]. The ALRs are modified bubble‐column reactors (BCRs) consist of three parts: riser, downcomer, and gas‐liquid separator [92]. However, ALRs are more suitable for shear‐sensitive biological processes as the flow in ALRs is organized in a defined cycle while it is random in BCRs.

**FIGURE 4:**
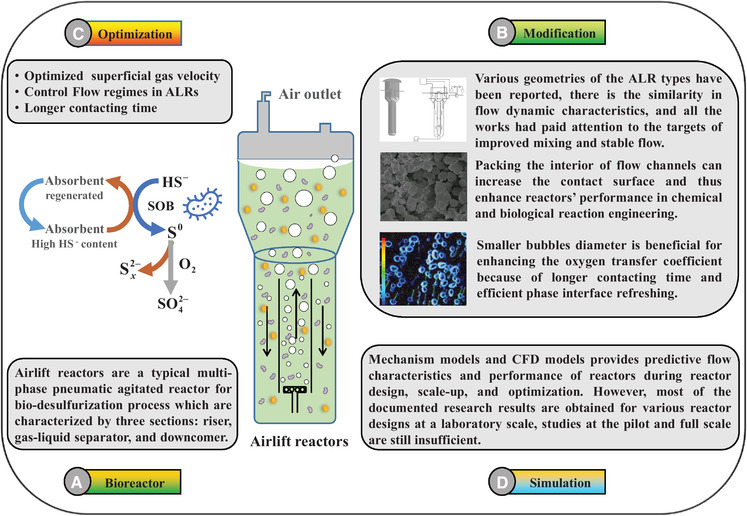
Schematic diagram summarizing the modification, optimization, and simulation of bioreactor. (A) A brief description of bioreactors. (B) Modify the bioreactor via the geometry, packing, and bubble diameter. (C) Three key parameters of bioreactor optimization. (D) Simulate the bioreactor via the mechanism model and computational fluid dynamics (CFD) model

The performance of ALRs is affected by multiple factors. In order to improve the performance of ALRs and get a deeper understanding of the regularities of scaling up, extensive research has been conducted in recent years. These studies were mainly covering the optimization of mass transfer by design and numerical simulation for flow dynamics characteristics in ALRs, as summarized in Figure 4.

### 5.1 Modifications and optimization of bioreactors

Although ALRs with various geometric have been reported, there are similarities in flow dynamic characteristics, and most of the previous studies focused on improving mass transfer and achieving stable flow. Unfortunately, a good mass transfer effect and a stable flow state are two contrasting targets of the flow uniformity of the risers. In this sense, the smaller bubble diameter can enhance the mass transfer and obtain a more stable fluid. The oxygen transfer is a key parameter in the aerobic biological process, as the aerobic bacteria need oxygen to grow, maintain cells, and form products [93]. The flow regime of the bubbles in the liquid phase is closely related to their size, the bubbles with a smaller diameter are easier to enter the downcomer and then circulate through the riser. As a result, the oxygen transfer coefficient can be improved for longer contact time and effective phase interface refresh [94].

The smaller bubbles diameter and longer contacting time can be obtained by some geometry modifiers like wire‐meshes, sieve plates, and static mixers to the risers of ALRs. Bun et al. developed a modified ALR by inserting the slanted baffles in the riser, which can maintain the bubble size between 3.88 and 4.63 mm within the studied superficial gas velocity range. This result is about 0.2 mm smaller than the average bubble diameter in the conventional reactor. As a result, the modified ALR can enhance the transfer coefficient value up to 97% compared to a regular bioreactor [95]. Furtherly, preliminary analysis of the flow dynamic characteristics and oxygen transfer parameters about this new reactor have been studied. Luo et al. introduced an ALR with sieve plates at different heights along the axial direction of the riser. The results showed that the sieve plates can break the large diameter bubbles and the gas holdup and the oxygen transfer coefficient are significantly increased by installing the sieve plate. To be specific, the values of gas holdup and the oxygen transfer coefficient were increased by more than 20% and 42% respectively at the superficial gas velocity (SGV) of 2.81 × 10^−3^ m/s [96].

The flow regime in ALR will change with the SGV, which will affect the hydrodynamics and mass transfer characteristics in the reactor. Typically, the flow regime in ALR may be defined following the distribution of bubbles in the bioreactor. At a low SGV, bubbles are mainly in the riser. With the increase of SGV, the flow regime in the riser gradually transits to turbulence, and more and more bubbles even enter the downcomer. To obtain a high mass transfer as well as a stable flow regime, an ALR with cross geometry inside can improve the flow characteristic in the riser, make the fluid flow quickly fluidization and get a good mixing effect at the same time [97].

Packing the interior of flow channels can enhance reactors’ performance in chemical and biological reaction engineering as it increases the contact area. In this sense, HS^−^ is biologically oxidized to S^0^ in an internal ALR via *Thioalkalivibrio versutus* under haloalkaliphilic conditions. So the formation of elemental S^0^ particles may enhance gas‐liquid mass transfer in the bioreactor just like adding particles to the bioreactor. The mechanism of the enhancement of the mixing effect by adding particles is that the particles change the thermodynamic relationship between the gas and liquid phases at the interface [98]. However, Tao et al. investigated the hydrodynamics and mass transfer (the influence of solid concentration, gas velocity, flow regime transition, and mass transfer) in a multistage ALR with a slurry system. The results showed that the increment of superficial gas velocity could increase the gas holdup, circulating liquid velocity, and volumetric mass transfer coefficient and at the same time decrease the mixing time in the gas‐liquid phase flow, while the opposite effects would get in gas‐liquid‐solid slurry flow [99].

Besides the hydrodynamic and mass transfer parameters, it is also a major challenge to effectively separate solids from ALRs, such as bio‐S^0^, to obtain clean circulating liquid [100]. a hydrocyclone and an ALR were successfully integrated for the liquid‐solid separation [88]. A pilot ALR combining separation and mixing was also studied by Geng et al. In that developed reactor, mixing, mass transfer, and liquid‐solid separation can be simultaneously realized without extra energy input. Moreover, the feasibility of the process intensification was firstly verified.[100]

Despite abundant research efforts, there is still a lack of universal theory to describe the flow dynamic characteristics of ALRs with various geometric and sizes. The design procedures of ALRs are usually empirical due to the complexity of the biochemical reactions and the multi‐phase flow in these reactors, and a comprehensive understanding of them is crucial for accurate design and scale‐up of ALRs. Therefore, a deep understanding of the detailed underlying flow mechanisms with different behaviors is the direction of future research.

### 5.2 Numerical simulation method

The design procedures of ALRs are usually empirical due to the complexity of the biochemical reactions and the multi‐phase flow in these reactors. Furthermore, the traditional experimental measurement methods have been widely employed in research and industry for reactor design, optimization, and scale‐up. However, traditional methods to reactor design need extensive experimental verification, which make them time‐consuming, laborious and expensive. Modeling methods can solve these problems. Modeling can provide predictable flow characteristics and reactor performance on the premise of construction. The main modeling methods of ALR include the mechanism model and CFD (computational fluid dynamics) model [94].

Mechanism models use simple mathematical theory to predict the hydrodynamic parameters in ALR. Furthermore, CFD modeling is based on solving Navier‐Stocks flow equations and turbulent vortex equation, which has made great progress in establishing the mathematical model and can be used in many applications [94]. However, most of the studies are carried out in various bioreactor designs at a laboratory scale, and pilot and full‐scale studies are still insufficient. Therefore, in the future investigation of the numerical simulation method of ALRs, a general scale‐up design rule is highly demanded to allow the design of ALR with high confidence.

## 6 CONCLUDING REMARKS

In this review, we have presented an overview of research progress on the biological gas desulfurization process, mainly involving the genetic modification of S^0^‐oxidizing bacteria, the enhancement of H_2_S absorption, and the modification, optimization, and simulation of bioreactors. Besides applied normally for gene editing of SOB, CRISPR‐Cas systems help develop desulfurizing biocatalysts with increased thermostability, wider substrate specificity, and increased solvent tolerance. Perhaps we need to develop efficient CRISPR‐Cas engineering methods for genetic modifications of industrial bacteria strains. And several enhancing ways of H_2_S absorption in an absorber urgently need to be studied further on an industrial scale to confirm whether they have a great effect on industrial H_2_S absorption. Lastly, modification of bioreactor and a full understanding of complex flow dynamics in various bioreactors are significant methods for reactor design, optimization, and scale‐up. In a word, better understanding and investigations of the BDS process is conducive to the implementation of high‐efficiency microbial capture of H_2_S and its industrial applications.

## CONFLICTS OF INTEREST

The authors have declared no conflicts of interest.

## Data Availability

Data sharing not applicable to this article as no datasets were generated or analysed during the current study.
